# Admissions to publicly-funded residential treatment for opioid use disorder and opioid toxicity outcomes after discharge in Ontario, Canada: A population-based repeated cross-sectional study

**DOI:** 10.1371/journal.pgph.0007566

**Published:** 2026-11-11

**Authors:** Nevena Rebić, Bisola Hamzat, Daniel McCormack, Paul Kurdyak, Shaleesa Ledlie, Zoë Dodd, Jennifer Wyman, Dana Shearer, Jes Besharah, Charlotte Munro, Jase Watford, Anjie Huang, Pamela Leece, Tara Gomes

**Affiliations:** 1 Ontario Drug Policy Research Network (ODPRN), St. Michael’s Hospital Research Institute, Toronto, Ontario, Canada; 2 St. Michael’s Hospital Research Institute, MAP Centre for Urban Health Solutions, Toronto, Ontario, Canada; 3 ICES, Toronto, Ontario, Canada; 4 Institute for Health Policy, Management and Evaluation, University of Toronto, Toronto, Ontario, Canada; 5 Department of Family and Community Medicine, University of Toronto, Toronto, Ontario, Canada; 6 Dalla Lana School of Public Health, University of Toronto, Toronto, Ontario, Canada; 7 Public Health Ontario, Toronto, Ontario, Canada; 8 Leslie Dan Faculty of Pharmacy, University of Toronto, Toronto, Ontario, Canada; PLOS: Public Library of Science, UNITED STATES OF AMERICA

## Abstract

With Canadian provinces expanding investments for bed-based inpatient (i.e., residential) substance use treatment, it’s important to understand who accesses these programs and their treatment outcomes. Therefore, the aim of our study was to describe admissions to publicly-funded residential treatment for opioid use disorder (OUD) and opioid toxicities rates following discharge. We conducted a population-based repeated cross-sectional study of publicly-funded residential treatment for OUD in Ontario, Canada, between July 1, 2013 and June 30, 2022. We calculated population-adjusted residential treatment admission rates and used joinpoint regression models to assess significant changes in monthly percent changes (MPC). We reported characteristics of discharged clients and weekly rates of fatal or non-fatal opioid toxicity 1-year post-discharge. Among the first identified residential treatment episode for each client, we determined the proportion who had an opioid toxicity event within 28-days post-discharge, overall and stratified by admission date, age, sex, mandated treatment, and discharge reason. Over the study period, admissions fluctuated monthly (from 1.5 [N = 18] to 11.9 [N = 136] per 1,000,000), declining gradually until December 2019 (MPC: -0.3%; p-value = 0.0009) before dropping abruptly between January and April 2020 (MPC: -20.3%; p-value = 0.44) and then rising soon thereafter (MPC, 2.5%; p-value = 0.0001). Overall, 60.8% of clients (N = 9,388; mean age, 33.1 + 10.2 years; 64.1% male) completed their treatment program. Within 28 days post-discharge, 1.4% experienced an opioid toxicity, with higher rates among clients who were male (1.7%), mandated to treatment (2.1%), and discharged early, both voluntarily (2.0%) and involuntarily (2.8%). Despite rising investments into residential treatment programs, our findings showed admissions for OUD dropped substantially early in the COVID-19 pandemic and remained below pre-pandemic levels in the subsequent two years. Importantly, high rates of early discharge and opioid toxicity soon thereafter underscore the need to implement quality standards for residential treatment programs in Ontario and throughout Canada.

## Introduction

The increased toxicity of Canada’s unregulated drug supply has claimed 52,544 lives since January 2016 and is largely driven by fentanyl and its analogues, which contributed to 79% of opioid-related deaths in 2024 [1]. While people with opioid use disorder (OUD) represent approximately 0.5% of the Canadian population [2], they account for nearly two-thirds of opioid-related deaths in Ontario [3].

Medications for opioid agonist treatment (OAT), such as methadone and buprenorphine/naloxone, have been recommended as first-line standard of care for OUD by national Canadian guidelines [4–6] since March 2018; however, the robust evidence base supporting their safety and effectiveness had been established over the preceding decades. People seeking more intensive support may access publicly or privately-funded inpatient residential treatment services, which are intensive structured live-in programs that typically last ≥4 weeks, may include pharmacologic (i.e., OAT) and non-pharmacologic interventions (e.g., individual and group counselling, self-help and life skills training, and support for community reintegration), and require clients to abstain from substance use during their stay [7–9]. However, the lack of consistent quality standards, policies, and performance metrics for these programs has resulted in inconsistent service delivery across Canada [10–12].

Canadian guidelines emphasize the limited available research evaluating the safety and effectiveness of residential treatment [4,5] and the need for programs to integrate concomitant OAT [4,13], as studies from Europe [14,15] show return to substance use is common following discharge from abstinence-based programs. A 2019 systematic review [16] of residential treatment outcomes found most studies had notable methodologic weaknesses as many employed small selective samples, relied on self‑reported measures with limited follow‑up, and showed high attrition due to incarceration, hospitalization, death, or loss of contact. Moreover, key features of residential treatment programs, including their specific content and how they are delivered, were often poorly described, restricting the generalizability and replicability of findings and translation of effective interventions into practice [16]. None of the included studies evaluated residential treatment outcomes using Canadian population-based administrative health data. Importantly. cohort studies from the United States [17], Sweden [18], and Australia [19], found an increased risk of mortality following residential treatment, with the highest number of deaths within the first 4 weeks following discharge, primarily due to suicide and overdose.

Despite the contentious position of residential treatment programs in OUD care, several Canadian provinces, including Ontario, have made substantial investments to expand these programs in recent years [20–23], including exploring the use of involuntary treatment [24–26]. Therefore, there is a pressing need to address existing knowledge gaps concerning the use and safety residential treatment for OUD as well as the lack of relevant Canadian research. As a first step, we aimed to describe trends in residential treatment admissions for OUD, including rates of admissions, the characteristics of admitted individuals and the treatment they received, and the risk of opioid toxicities following discharge, in Ontario, Canada.

## Methods

We conducted a population-based repeated cross-sectional study of publicly-funded residential treatment for OUD between July 1, 2013 and June 30, 2022 in Ontario, Canada.

### Ethics Statement

ICES is a prescribed entity under Ontario’s Personal Health Information Protection Act (PHIPA). Section 45 of PHIPA authorizes ICES to collect personal health information, without consent, for the purpose of analysis or compiling statistical information with respect to the management of, evaluation or monitoring of, the allocation of resources to or planning for all or part of the health system. Projects that use data collected by ICES under section 45 of PHIPA, and use no other data, are exempt from Research Ethics Board review. The use of ICES data in this study is authorized under section 45 and approved by ICES’ Privacy and Legal Office.

### Data sources

We used linked population-based health administrative databases housed at ICES (S1 Table). In 2023, the Drug and Alcohol Treatment Information System (DATIS), which contains clinical and administrative information on all Ontario patients admitted to the over 200 publicly-funded substance use programs since 1994 [27,28], was linked to the ICES data repository, creating a unique opportunity to conduct robust, population-based quantitative evaluations of residential treatment programs in Ontario. We used DATIS to identify all residential treatment admissions for OUD and to obtain client and service characteristics for each admission.

We used the Registered Persons Database to obtain demographic information and vital statistics, and the Narcotics Monitoring System (available since July 2012) and the Ontario Drug Benefit database, when earlier data was required, to identify all claims for controlled substances dispensed from community pharmacies. We identified emergency department (ED) visits, inpatient and mental health admissions using the Canadian Institute for Health Information’s National Ambulatory Care Reporting System, Discharge Abstract Database, and Ontario Mental Health Reporting System, respectively, and outpatient visits using the Ontario Health Insurance Plan Claims database. Finally, we used the Drug and Drug/Alcohol Related Deaths database to identify confirmed opioid toxicity deaths. All datasets were linked using unique coded identifiers and analyzed at ICES.

### Population Definition

We identified all admissions to a publicly-funded bed-based inpatient substance use service (i.e., residential treatment) during the study period where the primary substance of concern at intake was an opioid (prescription or non-pharmaceutical). We excluded short-term admission for withdrawal management (i.e., detox services), which typically last up to 7 days and assist with immediate withdrawal symptoms after ceasing or reducing opioid use [8], and extended stays for residential supportive treatment, which provide longer-term, lower-intensity, substance-free accommodations to assist with reintegration into the community [29,30], as these bed-based services differ from residential treatment in their duration, therapeutic intensity, and goals of care.

Our population was restricted to Ontario residents’ aged ≥15 years to reflect the population who typically engages in OUD treatment (i.e., community-based OAT) in Ontario [31]. As each client may be administered multiple residential treatment services, with or without other consecutive bed-based services (i.e., withdrawal management, residential supportive treatment, or medical/psychiatric treatment), during a single trajectory (referred to as an ‘episode’) of care, we defined the index date as the first date of a residential treatment service during a single episode of care and included all identified episodes for our analysis of admission trends. For our analyses of client characteristics and opioid toxicity trends, we restricted our population to individuals discharged to the community by excluding episodes with >1 residential treatment service record, missing discharge date, and death as reason for discharge. Finally, for our analysis of opioid toxicities 28 days post-discharge, we further restricted our population to the first residential treatment episode for each unique client.

### Characteristics

Demographic characteristics included sex, age, location of residence, neighborhood income quintile, employment status, and legal status at index (**S2 Table**). For comorbidities, we defined mental health-related diagnoses using information from outpatient physician and hospital visits in the previous 5 years as well as self-reported non-medical intravenous drug use (i.e., never injected, injected in the last 12-months, injected more than 12 months ago). We captured prior prescribing of OAT (i.e., buprenorphine-containing products, methadone, and slow-release oral morphine [SROM]), non-OAT opioids, benzodiazepines, and stimulants in the 30 days and 1 year prior to index. We also assessed indicators of healthcare utilization (i.e., any opioid-related ED visit or hospitalization; number of non-OUD outpatient physician visits, all-cause ED visits, and hospitalizations) in the 1 year prior to index. Finally, we captured characteristics of each residential treatment episode, including other presenting substances of concern (i.e., stimulants, alcohol, cannabis, tobacco, benzodiazepines, barbiturates), whether treatment was mandated and by whom (i.e., legal system/courts, child protective services, employer or school authority, family), reason for discharge (i.e., completed program, voluntary or involuntary early discharge), length of stay, and other bed-based services received prior to admission and following residential treatment discharge.

### Outcome

Our primary outcome was non-fatal or fatal opioid toxicity, defined as an inpatient hospitalization or ED visit for opioid toxicity using International Classification of Diseases, 10th revision (ICD-10) diagnosis codes (T40.0–T40.4, T40.6) or opioid-related death using coronial records. We observed each residential treatment episode from discharge for 365 days or until non-opioid related death, readmission (i.e., a subsequent episode of care), or maximum follow-up date of June 30, 2023, whichever came first.

### Analyses

We used Statistics Canada’s population estimates [32] to define Ontario population denominators and calculated crude annual and monthly population-adjusted rates of residential treatment admissions (per 1,000,000 population) over the study period. In an exploratory analysis, we investigated significant changes in admission rates, including around the time of the COVID-19 pandemic, using logarithmic joinpoint regression models to identify points where the trend significantly changes and selected the simplest model the data allow by comparing the weighted Bayesian Information Criterion values [33]. For each linear trend segment, we calculated monthly percent change (MPC) and corresponding 95% confidence intervals (CIs).

In a secondary analysis exploring the prevalence of integrated OAT in residential treatment programs, we determined the proportion of residential treatment episodes where clients were dispensed OAT (i.e., methadone, buprenorphine-containing products, or SROM) with a days’ supply that overlaps with the 7 days prior to admission or the 7 days after discharge among the subgroup of clients discharged from a single residential treatment service. Additionally, we calculated the annual population-adjusted rates of residential treatment admissions stratified by OAT exposure following discharge.

For our analysis of patient characteristics within the abovementioned subgroup, we used descriptive statistics overall and stratified by date of admission (i.e., July 1, 2013 to December 31, 2017; January 1, 2018 [study period midpoint] to June 30, 2022) to assess changes over time with an absolute standardized differences (StD) of >0.10 indicating a meaningful difference [34]. Then, for our analysis of opioid toxicity trends, we calculated weekly rates (per 100) of non-fatal or fatal opioid toxicities among uncensored individuals at the beginning of each weekly interval over 1 year of follow-up after residential treatment discharge.

Finally, to assess the differential risk at the time of the first residential treatment episode discharge for each unique client, we determined the proportion of people experiencing an opioid toxicity event within 28 days following discharge, overall and stratified by admission date, age, sex, voluntary/mandated treatment, and discharge reason. We compared proportions across strata using chi-square tests with a type I error rate of 5%. This study is reported as per the Reporting of Studies Conducted using Observational Routinely-Collected Data (RECORD) checklist [35].

## Results

We identified 9,948 publicly-funded residential treatment admissions for OUD during our study period (Fig 1), which included 9,388 residential treatment episodes among 7,729 unique clients who were discharged from a single residential treatment service.

**Fig 1:**
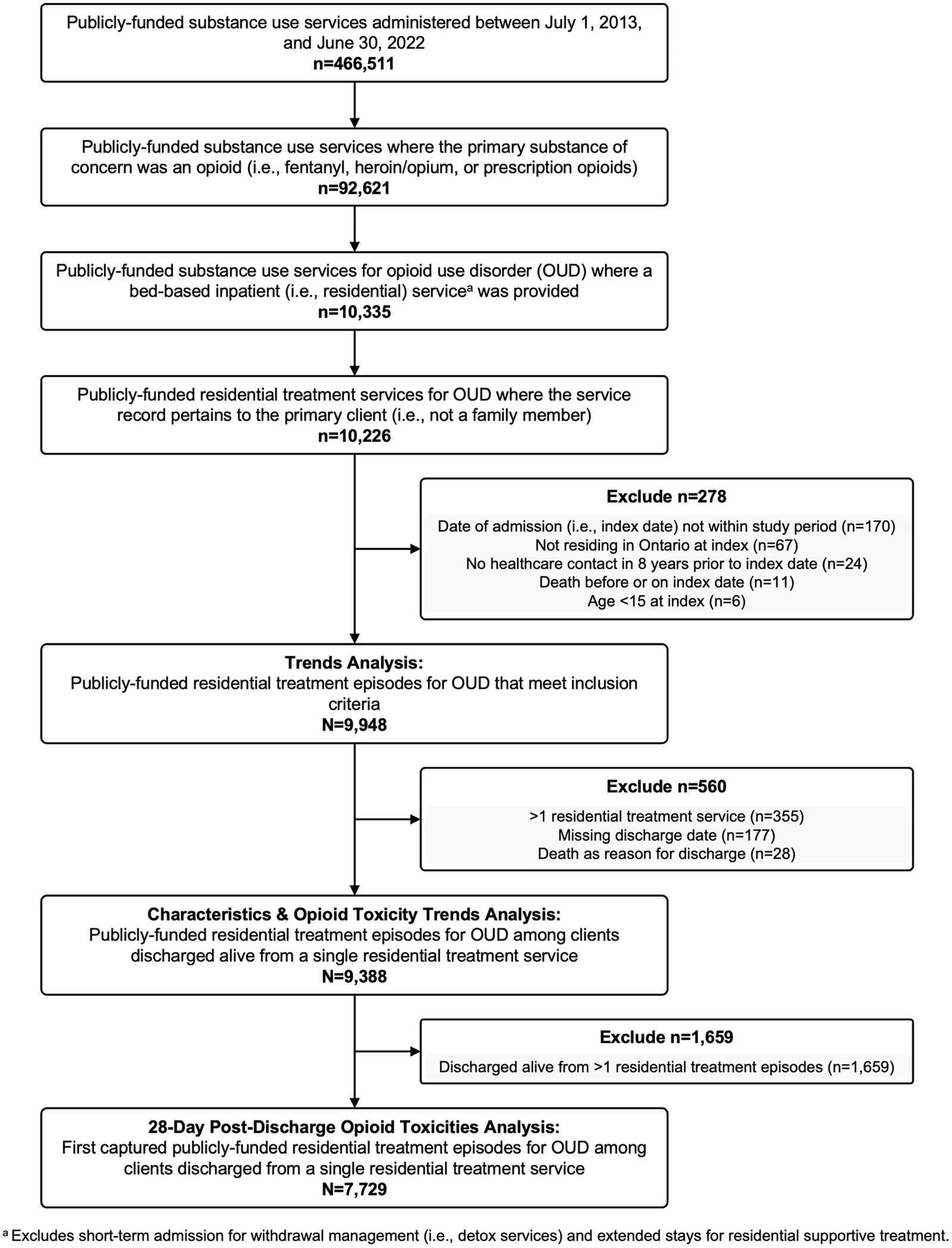
Flow diagram of population selection.

### Trends in residential treatment admissions

Among all publicly-funded residential treatment admissions, annual rates fell from 114.5 per 1,000,000 in 2014 to 72.7 per 1,000,000 in 2021 (S3 Table). Monthly residential treatment admission rates fluctuated over time, ranging from 1.5 to 11.9 per 1,000,000, with declines in December of each year (Fig 2). Our exploratory analysis showed that monthly admission rates declined slowly until December 2019 (MPC: -0.3%; 95% CI: -0.5%, -0.2%; S4 Table), followed by a rapid decline (MPC: -20.3%; 95% CI: -55.2%, 41.8%) between January and April 2020, coinciding with the start of the COVID-19 pandemic. Rates began to rise again soon thereafter (MPC, 2.5%; 95% CI: 1.2%, 3.7%) but remained below those seen prior to COVID-19 at the end of the study period.

**Fig 2:**
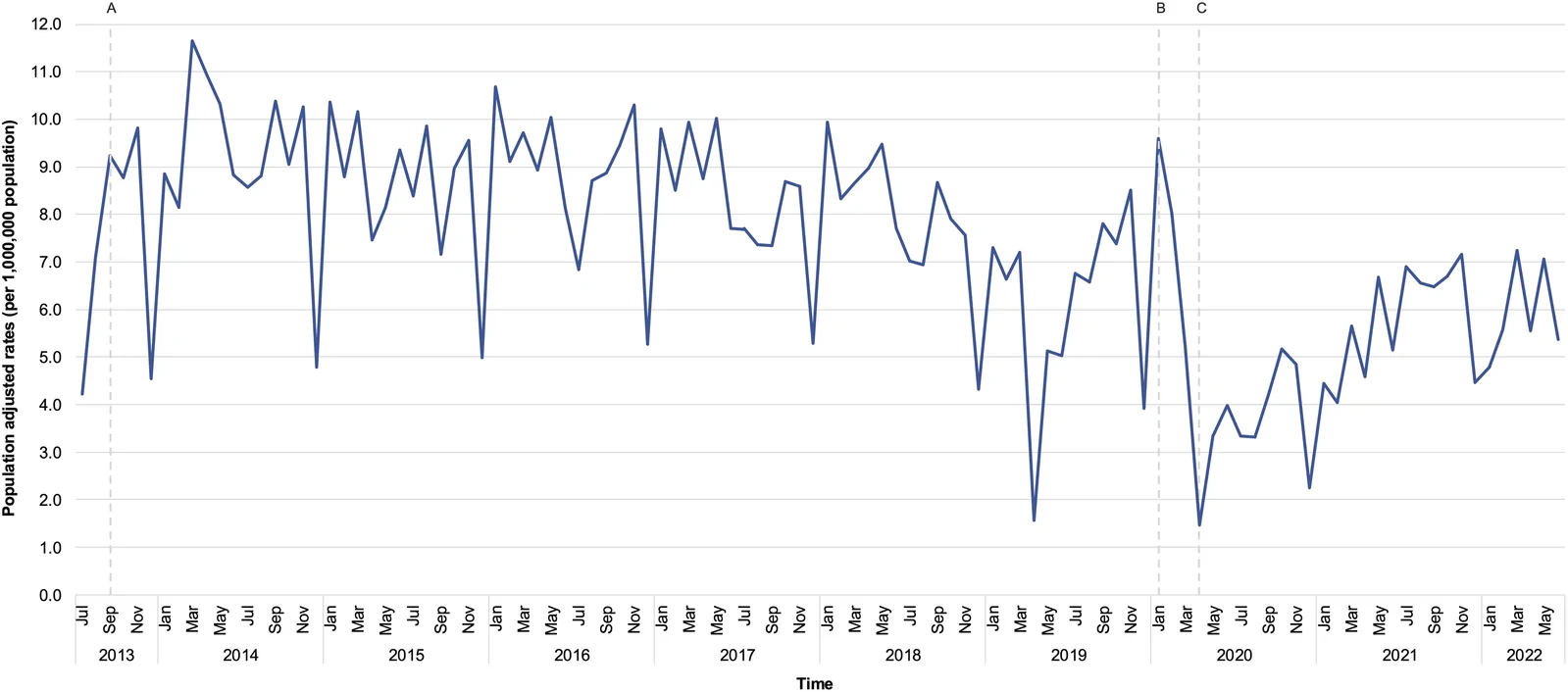
Monthly population-adjusted rate of all residential treatment admissions for OUD (N = 9,948) between July 1, 2013 and June 30, 2022. Note: vertical lines represent the three points (A, B, C) identified through jointpoint modeling of admission trends.

A secondary analysis of trends in residential treatment episodes among discharged clients from a single residential treatment service (N = 9,388) showed that just under one-third of all episodes were dispensed OAT within 7 days of admission (N = 2,887; 30.8%) as well as within 7 days of discharge (N = 2,824; 30.1%). Among those who did receive OAT before admission, the vast majority (88.9%; N = 2,567) were dispensed OAT following discharge; whereas, among those who did not receive OAT prior to admission, only 3.9% (N = 257) received OAT following discharge. Early in the study period, the rate of individuals discharged from residential treatment without receiving OAT in the week after discharge was 5 times greater than those who did receive OAT (annual rate of 91.7 compared to 19.0 per 1,000,000 population in 2014; S5 Table); however, this difference decreased over time, reaching similar rates by the start of the COVID-19 pandemic (S1 Fig).

### Population and service characteristics

Among all residential treatment episodes that resulted in discharge from a single residential treatment service, clients had a mean age of 33.10 ± 10.17, were predominately male (64.1%), with most residing in urban neighbourhoods (87.7%) and Southern Ontario (85.2%), and just under half were unemployed (48.8%; Table 1). While a majority (66.7%) did not have any legal issues, 16.9% were awaiting trial or sentencing and 12.0% were on probation or parole. Compared to clients admitted in the first half of the study period, receipt of OAT 30 days before admission was higher among those admitted after the study midpoint (25.5% vs. 46.9%; StD = 0.46).

**Table 1:** Characteristics of individuals discharged from a residential treatment episode for OUD, overall and stratified by date of admission.

Characteristics	All episodes (N = 9,388)	Date of admission before January 1^st^, 2018 (N = 5,372)	Date of admission on or after January 1^st^, 2018 (N = 4,016)	Standard Difference^a^
**DEMOGRAPHICS**				
**Age**, mean (SD)	33.10 + 10.17	32.60 + 10.8	33.76 + 9.85	0.12*
**Age**, N (%)				
15-18	333 (3.5%)	214 (4.0%)	119 (3.0%)	0.06
19-24	1,583 (16.9%)	1,062 (19.8%)	521 (13.0%)	0.18*
25-34	3,964 (42.2%)	2,215 (41.2%)	1,749 (43.6%)	0.05
35-44	2,169 (23.1%)	1,068 (19.9%)	1,101 (27.4%)	0.18*
45-54	956 (10.2%)	602 (11.2%)	354 (8.8%)	0.08
55+	383 (4.1%)	211 (3.9%)	172 (4.3%)	0.02
**Sex**, N (%)				
Male	6,019 (64.1%)	3,482 (64.8%)	2,537 (63.2%)	0.03
Female	3,369 (35.9%)	1,890 (35.2%)	1,479 (36.8%)	0.03
**Location of residence**, N (%)				
Urban	8,234 (87.7%)	4,725 (88.0%)	3,509 (87.4%)	0.02
Rural	1,054 (11.2%)	600 (11.2%)	454 (11.3%)	0.00
Missing	100 (1.1%)	47 (0.9%)	53 (1.3%)	0.04
**Location of residence in Ontario**, N (%)				
Southern Ontario	7,994 (85.2%)	4,616 (85.9%)	3,378 (84.1%)	0.05
Northern Ontario	1,392 (14.8%)	754 (14.0%)	638 (15.9%)	0.05
Missing	1-5	1-5	0	0.03
**Neighbourhood income quintile**, N (%)				
Lowest	3,091 (32.9%)	1,799 (33.5%)	1,292 (32.2%)	0.03
Medium-low	2,108 (22.5%)	1,185 (22.1%)	923 (23.0%)	0.02
Middle	1,610 (17.1%)	926 (17.2%)	684 (17.0%)	0.01
Medium-high	1,294 (13.8%)	731 (13.6%)	563 (14.0%)	0.01
Highest	1,175 (12.5%)	677 (12.6%)	498 (12.4%)	0.01
Missing	110 (1.2%)	54 (1.0%)	56 (1.4%)	0.04
**Employment status**, N (%)				
Unemployed (Looking for work)	4,581 (48.8%)	2,668 (49.7%)	1,913 (47.6%)	0.04
Employed full-time/part-time	2,199 (23.4%)	1,340 (24.9%)	859 (21.4%)	0.08
Disabled (not working)	1,276 (13.6%)	611 (11.4%)	665 (16.6%)	0.15*
Not in labour force (e.g., Homemaker)	884 (9.4%)	500 (9.3%)	384 (9.6%)	0.01
Student/Retraining	282 (3.0%)	170 (3.2%)	112 (2.8%)	0.02
Retired	74 (0.8%)	41 (0.8%)	33 (0.8%)	0.01
Unknown	92 (1.0%)	42 (0.8%)	50 (1.2%)	0.05
**Legal status**, N (%)				
None	6,261 (66.7%)	3,656 (68.1%)	2,605 (64.9%)	0.07
Awaiting trial or sentencing	1,588 (16.9%)	820 (15.3%)	768 (19.1%)	0.10
Probation/Parole	1,124 (12.0%)	689 (12.8%)	435 (10.8%)	0.06
Incarcerated	104 (1.1%)	52 (1.0%)	52 (1.3%)	0.03
Other	241 (2.6%)	122 (2.3%)	119 (3.0%)	0.04
Unknown	70 (0.7%)	33 (0.6%)	37 (0.9%)	0.04
**COMORBIDITIES**				
**Mental health diagnoses**^b^, N (%)				
Any mental health disorders (excluding SUD)	4,228 (45.0%)	2,301 (42.8%)	1,927 (48.0%)	0.10
Anxiety disorders	2,490 (26.5%)	1,345 (25.0%)	1,145 (28.5%)	0.08
Mood disorders	1,883 (20.1%)	1,060 (19.7%)	823 (20.5%)	0.02
Deliberate self-harm	1,729 (18.4%)	900 (16.8%)	829 (20.6%)	0.10
Schizophrenia and other psychotic disorder	601 (6.4%)	276 (5.1%)	325 (8.1%)	0.12*
Other mental health disorders	699 (7.4%)	343 (6.4%)	356 (8.9%)	0.09
**Non-medical intravenous drug use**, N (%)				
Never injected	4,517 (48.1%)	2,718 (50.6%)	1,799 (44.8%)	0.12*
Injected in the last 12 months	3,543 (37.7%)	1,925 (35.8%)	1,618 (40.3%)	0.09
Injected more than 12 months ago	1,030 (11.0%)	554 (10.3%)	476 (11.9%)	0.05
Unknown	298 (3.2%)	175 (3.3%)	123 (3.1%)	0.01
**PRIOR PRESCRIBING**				
**OAT prescription 30 days prior to index**, N (%)	3,252 (34.6%)	1,369 (25.5%)	1,883 (46.9%)	0.46*
Methadone	1,987 (21.2%)	914 (17.0%)	1,073 (26.7%)	0.24*
Buprenorphine	1,357 (14.5%)	490 (9.1%)	867 (21.6%)	0.35*
SROM	53 (0.6%)	0	53 (1.3%)	0.16*
**OAT prescription 1 year prior to index**, N (%)	4,539 (48.3%)	2,099 (39.1%)	2,440 (60.8%)	0.44*
Methadone	3,075 (32.8%)	1,501 (27.9%)	1,574 (39.2%)	0.24*
Buprenorphine	2,470 (26.3%)	955 (17.8%)	1,515 (37.7%)	0.46*
SROM	125 (1.3%)	1-5	122 (3.0%)	0.24*
**Prescribed medications 30 days prior to index**, N (%)				
Non-OAT opioids	645 (6.9%)	456 (8.5%)	189 (4.7%)	0.15*
Stimulants	500 (5.3%)	233 (4.3%)	267 (6.6%)	0.10
Benzodiazepines	1,772 (18.9%)	1,108 (20.6%)	664 (16.5%)	0.11*
**Prescribed medications 1 year prior to index**, N (%)				
Non-OAT opioids	2,712 (28.9%)	1,822 (33.9%)	890 (22.2%)	0.26*
Stimulants	922 (9.8%)	456 (8.5%)	466 (11.6%)	0.10
Benzodiazepines	3,613 (38.5%)	2,180 (40.6%)	1,433 (35.7%)	0.10
**HEALTHCARE UTILIZATION**				
**Opioid toxicity-related ED visit or hospitalization**^c^, N (%)	1,009 (10.7%)	309 (5.8%)	700 (17.4%)	0.37*
**Health system utilization** ^c^				
Number of outpatient physician visits (excluding OUD), median (IQR)	7 (3–15)	7 (3–14)	8 (3–17)	0.15*
Number of ED visits, median (IQR)	2 (1–4)	2 (1–4)	2 (1–5)	0.12*
Number of hospitalizations, mean + SD	0.26 ± 0.75	0.23 ± 0.69	0.30 ± 0.82	0.09

^a^Standard difference (StD) between stratified groups that are > 0.10 are indicated by an *.

^b^Measured 5 years prior to the index date.

^c^Measured 1 year prior to the index date.

**Definitions:** ED – emergency department; IQR – interquartile range; OAT – opioid agonist treatment; OUD – opioid use disorder; SD – standard deviation; SUD – substance use disorder.

Most clients (89.8%) presented with concurrent substance use, most commonly stimulants (64.7%), alcohol (45.0%), cannabis (41.4%), and tobacco (32.7%; Table 2). The proportion with a secondary concern with alcohol (50.8% vs. 37.2%; StD = 0.28) and cannabis (43.9% vs. 37.9%; StD = 0.12) was lower among clients admitted after the study midpoint, while those with stimulants (62.3% vs. 67.9%; StD = 0.12) and tobacco (30.4% vs. 35.7%; StD = 0.11) slightly increased. An important proportion (15.3%) of our cohort were mandated to residential treatment, primarily by the legal system and child protective services. While many clients completed the residential treatment program (60.8%), a substantial portion were voluntarily (21.3%) and involuntarily (15.4%) discharged early.

**Table 2:** Treatment characteristics among individuals discharged from a residential treatment episode for OUD, overall and stratified by date of admission.

Characteristics	Whole cohort (N = 9,388)	Date of admission before January 1^st^, 2018 (N = 5,372)	Date of admission on or after January 1^st^, 2018 (N = 4,016)	Standard Difference^a^
**Presenting problem substances (excluding opioids)**, N (%)	8,432 (89.8%)	4,842 (90.1%)	3,590 (89.4%)	0.02
Stimulants	6,071 (64.7%)	3,345 (62.3%)	2,726 (67.9%)	0.12*
Alcohol	4,223 (45.0%)	2,731 (50.8%)	1,492 (37.2%)	0.28*
Cannabis	3,882 (41.4%)	2,359 (43.9%)	1,523 (37.9%)	0.12*
Tobacco	3,067 (32.7%)	1,635 (30.4%)	1,432 (35.7%)	0.11*
Benzodiazepines	988 (10.5%)	614 (11.4%)	374 (9.3%)	0.07
Barbiturates	20 (0.2%)	12 (0.2%)	8 (0.2%)	0.01
**Mandatory/required treatment**, N (%)				
Not mandatory Treatment	7,855 (83.7%)	4,500 (83.8%)	3,355 (83.5%)	0.01
Mandatory/required Treatment	1,440 (15.3%)	842 (15.7%)	598 (17.8%)	0.02
*Legal system*	634 (6.8%)	372 (6.9%)	262 (6.5%)	0.02
*Child protective services*	254 (2.7%)	144 (2.7%)	110 (2.7%)	0.00
*Employee or school authority*	212 (2.3%)	132 (2.5%)	80 (2.0%)	0.03
*Family*	208 (2.2%)	131 (2.4%)	77 (1.9%)	0.04
*Other*	132 (1.4%)	63 (1.2%)	69 (1.7%)	0.05
Unknown/missing	93 (1.0%)	30 (0.6%)	63 (1.6%)	0.10
**Reason for discharge**, N (%)				
Completed Program	5,710 (60.8%)	3,278 (61.0%)	2,432 (60.6%)	0.01
Voluntary Discharge	1,997 (21.3%)	1,160 (21.6%)	837 (20.8%)	0.02
Involuntary Discharge	1,444 (15.4%)	811 (15.1%)	633 (15.8%)	0.02
Other	208 (2.2%)	116 (2.2%)	92 (2.3%)	0.01
Unknown/Missing	29 (0.3%)	7 (0.1%)	22 (0.5%)	0.07
**Length of Stay**, median (IQR)	20 (13–28)	21 (13–28)	20 (12-29)	0.03
**Services received prior to residential treatment admission**, N (%)				
Withdrawal management	3,457 (36.8%)	2,140 (39.8%)	1,317 (32.8%)	0.15*
Residential supportive treatment	442 (4.7%)	227 (4.2%)	215 (5.4%)	0.05
Medical/psychiatric treatment	1-5	1-5	0	0.03
**Services received following residential treatment discharge**, N (%)				
Withdrawal management	690 (7.3%)	445 (8.3%)	245 (6.1%)	0.08
Residential supportive treatment	382 (4.1%)	223 (4.2%)	159 (4.0%)	0.01
Medical/psychiatric treatment	1-5	1-5	0	0.03

^a^Standard difference (SD) between stratified groups that are > 10 are indicated by an *.

**Definitions:** ED – emergency department; IQR – interquartile range; OAT – opioid agonist treatment; OUD – opioid use disorder; SD – standard deviation; SUD – substance use disorder.

### Opioid toxicity after discharge

Weekly rates of opioid-related toxicity were highest in the first (0.7%; N = 66) and second (0.4%; N = 39) week following discharge, after which weekly rates fluctuated between 0 and 0.3% over the 1-year follow-up period (Fig 3). In the first 28 days following discharge from each client’s first identified residential treatment episode, 1.4% (N = 109) of people experienced an opioid toxicity; however, this varied significantly across strata (Table 3). Specifically, 28-day rates of opioid toxicity were significantly higher among individuals admitted to treatment in 2018 onwards (2.3%) compared with those admitted prior (0.8%; p < 0.0001) and among males (1.7%) compared with females (0.9%; p = 0.002). Importantly, rates were also higher among those mandated to residential treatment (2.1%) compared to those who were not (1.3%; p = 0.06), and among those voluntarily (2.0%) and involuntarily (2.8%) discharged early compared to those who completed their intended treatment program (0.09%; p < 0.0001) (Table 3).

**Fig 3:**
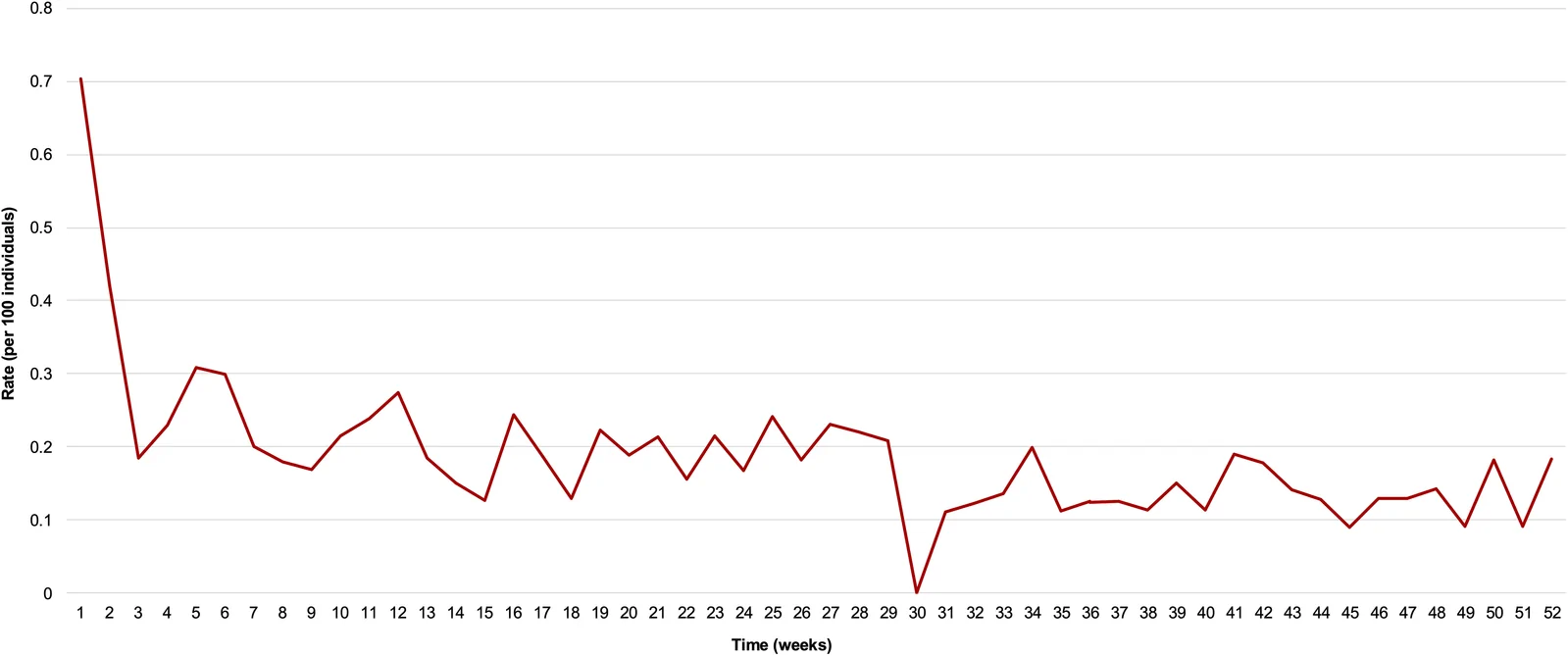
Weekly rate of non-fatal or fatal opioid-related toxicity events in the 1 year following discharge among individuals discharged from a residential treatment episode for OUD (N = 9,388).

**Table 3:** Total and proportion of fatal and non-fatal opioid toxicity in the first 28 days following residential treatment discharge for OUD, overall and stratified by key demographic and treatment characteristics.

Characteristics	Total discharge	Total with opioid toxicity	Percentage (%)	P-value^a^
**N**	7,729	109	1.4	
**Date of admission**, (N, %)				
Before January 1st, 2018	4,645	39	0.8	<0.0001*
On or after January 1st, 2018	3,084	70	2.3	
**Age**, (N, %)				
15-18	296	1-5	0.3-1.7^b^	0.26
19-24	1,322	23	1.7	
25-34	3,184	52	1.6	
35-44	1,788	20	1.1	
45-54	811	10	1.2	
55+	328	1-5	0.3-1.5^b^	
**Sex**, (N, %)				
Male	4,835	84	1.7	0.002*
Female	2,894	25	0.9	
**Mandated Treatment**, (N, %)				
Non-mandatory	6,441	82	1.3	0.06
Mandatory	1,214	26	2.1	
**Reason for Discharge**, (N, %)				
Completed Program	4,752	41	0.9	<0.0001*
Voluntary Discharge	1,631	32	2.0	
Involuntary Discharge	1,151	32	2.8	
Other/Unknown	195	1-5	0.5-2.6^b^	

^a^P-value calculated using chi-square test with those > 0.05 indicated by an *.

^b^Due to small cell counts, this estimate is reported as a range.

**Definitions:** ED – emergency department; IQR – interquartile range; OAT – opioid agonist treatment; OUD – opioid use disorder; SD – standard deviation; SUD – substance use disorder.

## Discussion

In our population-based study of publicly-funded residential treatment in Ontario, we found that residential treatment admission rates for OUD have progressively declined since mid-2013, with a notable disruption at the onset of the COVID-19 pandemic. Furthermore, admission rates remained below pre-pandemic levels by the end of the study period despite a rising trend beginning in mid-2020 and more recent investments into these programs [22,23]. All together, we observed fewer than 1,500 publicly-funded residential treatment stays for OUD annually between 2013 and 2022, while Ontarians accessing OAT rose from 45,917 (3.4 per 1,000 population) to 67,752 (4.5 per 1,000 population) individuals over the same period [31]. It is notable that only about 60% of residential treatment admissions for OUD resulted in program completion and just under a third received OAT immediately after discharge. Collectively, these findings raise questions regarding the capacity and capability of these programs to address the ongoing rise in opioid-related harms nationally. With 1 in 71 people experiencing an opioid toxicity within one month of leaving residential treatment, and even higher rates among people mandated to treatment and those discharged early, our findings additionally signal an urgent need to better manage OUD care during this transitional period.

Our findings are consistent with prior international studies of mortality following discharge from residential treatment programs. A United States [36] study of New York City reported lower rates of drug-related death in the first 14 days following termination of outpatient treatment (adjusted hazard ratio [aHR], 0.24; 95% CI, 0.12–0.48), medically supervised withdrawal management (aHR, 0.30; 95% CI, 0.16–0.56), and OAT (aHR, 0.47; 95% CI, 0.27–0.84) compared to discharge from residential treatment, with similar trends observed over a 90-day period following termination of these services. A similar study [17] study of drug-related mortality after the discontinuation of at least one the above mentioned substance-related services in the state of Texas reported significantly higher rates of drug-related deaths in the first 28 days following discharge from residential treatment (5.6 per 1,000 person years [PY]) compared to later periods over a 1-year follow-up (2.9 and 2.7 per 1,000 PY 29–90 and 91–365 days post-discharge, respectively). Higher 28-day mortality following a substance-related service (28.2% of which were for residential treatment) was also associated with use of opioids (adjusted odds ratio [aOR], 2.5; 95% CI, 1.4–4.4) and inversely associated with females (aOR 0.6; 95% CI, 0.4–0.8) and treatment completion (aOR 0.6; 95% CI, 0.4–0.9) [17]. Likewise, our study showed a higher proportion of opioid-related toxicities following residential treatment among clients who were male and did not complete treatment, particularly when discharge was involuntary. Another important demographic that warrants consideration is individuals who have been mandated to residential treatment, when they otherwise would not have chosen to be admitted, due to the recent resurgence of this practice in Canada [24–26]. A Swedish study of mandated residential treatment for substance use found high all-cause mortality (7.1 per 100 PY) in the year following discharge and a higher risk of death from external causes, mainly drug poisoning, in the first 2 weeks compared to remaining follow-up time (HR, 2.6; 95% CI, 1.3–5.0), particularly among males and younger individuals [18]. In line with these findings, we found higher rates of opioid toxicity among those mandated to treatment (1 in 47 of which experienced an opioid toxicity within 28 days of discharge), suggesting a link between involuntarily treatment and increased risk of negative health outcomes, including overdose and death [18,37–39], which may be correlated with individuals’ engagement in treatment [40–42], the severity of substance use [43,44], and the criminalization of people who use drugs [44,45]. Similar higher rates of opioid-related death have been observed immediately following release from incarceration in Ontario, including among Indigenous and Black people, who are often over-represented in correctional facilities [46–48]. Indeed, the practice of mandating residential treatment for substance use has been described as a legacy of colonial oppression by many Indigenous, Black, and racialized people, that infringes on their safety and rights to self-determination [43].

Several factors may have contributed to the declining utilization of publicly-funded residential treatment programs over the study period. As early as 2019, Ontario’s Auditor General [11,12] reported misalignments between the regional allocation of provincial funding and local population needs resulting in a geographic maldistribution of available beds across Ontario [11,12], with one study finding a 7-fold difference between the best and worst-served regions [10]. Additionally, growing wait times, which rose from a provincial average of 43 to 50 days between the 2014/15 and 2018/19 fiscal years, have led to some individuals dropping off waitlists due to hospitalization, incarceration, or even death [11,12]. These system-level constraints have been met with a growth in private, for-profit residential treatment programs which often have short or non-existent wait times but may pose significant financial burdens to clients [10,29]. The inequities created by a two-tiered system where access depends on ability to pay in addition to the lack of accountability mechanisms to ensure the provision of quality, evidence-based care have been major critiques of these private programs [10,29]. At the same time, the availability of community-based OAT has expanded since the removal of federal regulation requiring health professionals to obtain a Health Canada exemption to prescribe methadone in 2018 [49,50] while the increasing dominance of fentanyl in the unregulated drug supply, for which methadone has demonstrated particular success in mitigating symptoms of withdrawal [51,52], may have shifted individuals seeking treatment towards OAT. In contrast to the growing uptake in community-based OAT in the general Ontario population [31], program policies and eligibility criteria restricting or precluding its use within residential care represent a growing barrier to accessing residential treatment for clients already receiving OAT. As of 2023, 1 in 8 residential treatment programs in Ontario did not accept clients taking any form of OAT and a further 1 in 4 programs did not accepting clients on methadone [10], with evidence of these policies being even more restrictive in prior years [53]. Finally, the release of national guidance harmonizing clinical standards for the management of OUD across Canada in March 2018 [4], which specifically positioned OAT as first-line treatment and characterized the evidence base for residential treatment as weak, likely contributed to the deepening troughs within the declining trend in monthly residential treatment admission rates observed in the two years preceding the onset of the COVID-19 pandemic.

It is important to contextualize our findings within the broader evidence base for OUD treatment. In line with national guidelines recommending that residential treatment be delivered with concurrent OAT to offset risk of opioid-related toxicity immediately following discharge [4,5], we observed the rate of clients who received OAT following residential treatment increase over time, with just over half of admitted individuals connected to OAT post-discharge by the end of the study period. However, fewer than 5% appeared to be newly initiated on OAT during their stay in residential treatment, signaling a potential missed opportunity to provide clients with evidence-based care in line with current guidelines [4,13]. Our findings build on prior research linking DATIS to program-level information of publicly-funded residential treatment for opioid use in Ontario that revealed 52.8% of patients were admitted to programs that allowed them to use OAT between 2013–2016, among which only two program provided access to an OAT prescriber onsite [54]. In the same study, 56.8% of patients completed their program, with 23.3% voluntarily leaving early and 17.0% involuntarily discharged [54], reflecting similarly troubling incompletion rates as our own findings. Restrictions on OAT use during residential treatment may reflect historical program philosophies favoring abstinence [10]. Equally, programs are often staffed primarily by addiction counsellors and support workers [10], which greatly limits their capacity to support OAT initiation or dose adjustments, let alone provide care to clinically complex patients such as those with severe and persistent concurrent mental health disorders [29]. Reassuringly, most clients who were dispensed OAT prior to admission to residential treatment in our study, also received it shortly following discharge. Nonetheless, structural barriers in clinical staffing and program eligibility, including OAT-related exclusion criteria, likely restrict the integration of evidence‑based pharmacotherapy and clients’ ability to access comprehensive OUD treatment options while receiving residential care. Coupled with high rates of early discharge from residential treatment, which have been linked to more severe substance use, greater cognitive dysfunction, and unstable living arrangements [55,56], our findings reveal the importance of developing quality standards that prioritize patient‑centred and accessible care models. These should include supporting patient’s self-directed treatment goals, addressing complex care needs (e.g., polysubstance use) and vulnerabilities (e.g., assistance with housing instability, criminal/legal system), facilitating connections to community support (e.g., increasing visitation opportunities, involving peer support specialists), and following the best available evidence, including access to OAT and other harm reduction strategies [57,58]. Moreover, for First Nations, Inuit, and Métis Peoples, who have been disproportionately affected by the opioid toxicity crisis in Canada [59], it is recognized that culturally grounded, holistic models of care that incorporate Indigenous knowledge, including traditional ceremonies and teachings, and focus on strengthening individuals’ connections with the land, culture, language, and community, which may have been strained due to trauma, are essential to providing culturally safe substance use care to this population [60,61].

Our study addresses an important gap in Canadian research evaluating residential treatment for OUD and provides novel population-level information on long-term outcomes following discharge facilitated by recent linkages between DATIS and administrative health databases at ICES. However, limitations of our study warrant comment. First, we were unable to include privately funded residential treatment programs; as these facilities are not mandated to report to DATIS, their data are often inaccessible. Second, as we excluded episodes with multiple substance use services to ensure we had an accurate discharge date of clients’ transition to community residence, this may limit the generalizability of our findings, although only 3.6% (N = 355) were excluded at this step. Third, as we were unable to capture non-fatal toxicities treated outside of hospital, the actual number of opioid toxicities post-discharge are likely higher than reported. Fourth, we could not account racial disparities in residential treatment access and outcomes as race-based data is not routinely collected in administrative health data. Finally, we did not have information on in-treatment provision of OAT or program procedures for discharge planning, which could influence opioid risk at discharge. As adoption of provincial standards for organizations providing residential treatment in Ontario is voluntary, without meaningful mechanisms for enforcement, there is considerable variability in the quality and delivery of these programs related to their treatment philosophy (i.e., abstinence-based or harm-reduction orientation), staffing models and availability of OAT prescribers, policies regarding OAT initiation and continuation, discharge planning practices, provision of aftercare, and integration with community-based services [10–12,62,63]. Heterogeneity across these dimensions likely impacted rates of program completion and patient outcomes following discharge as well as comparisons between administering programs. As such, further research is needed to account for impact on program-level factors and other confounders on the effectiveness and safety of residential treatment for OUD.

Ultimately, our study provides critical evidence about who accesses publicly-funded residential treatment for OUD in Canada and their risk of toxicity following discharge, information that is increasingly urgent as investments into these programs expand across the country [20–23]. Together, our findings highlight a critical need for systemic changes imposing standards that embed patient‑centred and culturally safe practices, support safer transitions in care, and reduce punitive or abstinence‑only approaches, including the use of legally mandated treatment for OUD [43]. Continued evaluation of these programs remains imperative, starting with facilitating more expansive data collection on the care and services provided to clients, including OAT, across publicly-funded and private residential treatment programs.

## Supporting information

S1 Table Description of all linked databases used in study. (PDF)

S2 Table Data sources and variable definitions. (PDF)

S3 Table Annual counts and population adjusted rates of all residential treatment admissions for OUD (N = 9,948) between July 1, 2013 and June 30, 2022. (PDF)

S4 Table Estimated monthly percent changes (MPC) of all residential treatment admissions for OUD (N = 9,948) between July 1, 2013 and June 30, 2022. (PDF)

S5 Table Annual totals and population adjusted rates of residential treatment admissions among individuals discharged from a residential treatment episode for OUD (N = 9,388) between July 1st, 2013 and June 30th, 2022, overall and stratified by whether OAT was received in the 7 days following discharge from residential treatment. (PDF)

S1 Fig Monthly population adjusted rates of residential treatment admissions among individuals discharged from a residential treatment episode for OUD (N = 9,388) between July 1st, 2013 and June 30th, 2022, overall and stratified by whether OAT was received in the 7 days following discharge from residential treatment. (PDF)
