# Supplementary material for: Admissions to publicly-funded residential treatment for opioid use disorder and opioid toxicity outcomes after discharge in Ontario, Canada: A population-based repeated cross-sectional study
Source: PLOS Glob Public Health. 2026 Nov 11;6(11):e0007566. doi: 10.1371/journal.pgph.0007566 (PMC13754215; doi:10.1371/journal.pgph.0007566)
Supplement: Supplementary file 1 [file pgph.0007566.s001.pdf]

S1 Table. Description of all linked databases used in study.

Database	Description
CIHI Discharge Abstract Database (DAD)	Captures information on diagnoses and procedures for all inpatient hospital stays in Ontario.
CIHI National Ambulatory Care Reporting System (NACRS)	Captures information on diagnoses and procedures for all emergency department visits in Ontario.
CIHI Ontario Mental Health Reporting System (OMHRS)	Captures information on diagnoses and procedures for all inpatient stays in mental health hospitals in Ontario.
Drug and Alcohol Treatment Information System (DATIS)	Captures clinical and administrative information on all patients admitted to publicly funded addiction service programs in Ontario.
Drug and Drug/Alcohol Related Deaths (DDARD)	Captures information from investigations of all sudden and unexplained deaths in Ontario where an investigating coroner has determined that opioids alone or in the presence of other substances were direct contributors to death.
Narcotics Monitoring System (NMS)	Captures information on all prescriptions for controlled substances dispensed from community pharmacies in Ontario, regardless of payer.
Ontario Drug Benefit Database (ODB)	Captures all prescription drug claims for people covered by the ODB program, which consists of older adults, those aged <25 without private insurance, people on social assistance, and those with high drug costs relative to income.
Ontario Health Insurance Plan (OHIP) Database	Captures information on physician services provided using billing information for services covered by OHIP in Ontario.
Postal Code Conversion File (PCCF+)	Dataset allows for matching of six-digit postal codes to census geographies. Contains area-level equity stratifies for income quintiles, geography (rural vs. urban), and census metropolitan areas.
Registered Persons Database (RPDB)	Captures demographic and vital status characteristics for everyone eligible for the publicly funded Ontario Health Insurance Plan (OHIP) in Ontario.
Statistics Canada Census Data	Publicly available data from Statistics Canada used to identify population denominators.

Definitions: CIHI – Canadian Institute for Health Information
