# Supplementary material for: Admissions to publicly-funded residential treatment for opioid use disorder and opioid toxicity outcomes after discharge in Ontario, Canada: A population-based repeated cross-sectional study
Source: PLOS Glob Public Health. 2026 Nov 11;6(11):e0007566. doi: 10.1371/journal.pgph.0007566 (PMC13754215; doi:10.1371/journal.pgph.0007566)
Supplement: Supplementary file 2 [file pgph.0007566.s002.pdf]

S2 Table. Data sources and variable definitions.

Variable	Data sources	Definition
**DEMOGRAPHIC AND CLINICAL CLIENT CHARACTERISTICS**		
Age	RPDB	Age at index. Grouped as: 15-18, 19-24, 25-34, 35-44, 45-54 and 55+ years.
Sex	RPDB	Grouped as: Male, Female.
Location of residence	RPDB	Rurality of residence using postal code information. Grouped as: Rural, Urban.
Location of residence in Ontario	RPDB	Location of residence using postal code information. Grouped as: Northern Ontario, Southern Ontario.
Neighborhood income quintile	RPDB	Neighborhood income quintile using postal code information. Grouped as: Lowest, Medium-low, Middle, Medium-high, Highest.
Employment status	DATIS	Captures client's primary relation to the labour force in the three months prior to residential treatment admission. Grouped using the following predefined: Unemployed (Looking for work), Employed full-time/part-time (includes self-employed), Disabled (not working), Student/Retraining, Not in labour force (e.g., homemaker), Retired, Unknown.
Legal status	DATIS	Indicates involvement in the criminal justice system or youth justice system at the time of residential treatment admission. Grouped using the following pre-defined categories: No problem, Awaiting trial or sentencing, Probation, Parole Incarcerated, Other, Unknown.
Mental health-related diagnosis	DAD (ICD-10), NARCS (ICD-10), OMHRS (ICD-9)	Any hospital visit for mental health-related diagnosis in the prior 5 years (excluding SUD diagnosis). Grouped as: Any mental health disorder, Anxiety disorder, mood disorder, deliberate self-harm, Schizophrenia and other psychotic disorder, other mental health disorders. Defined using validated definitions.[1]
Non-medical intravenous drug use	DATIS	Grouped as: Never injected, Injected in the last 12-months, Injected more than 12 months ago.
Prescriptions for OAT prior to index	NMS	Defined as any methadone or buprenorphine or slow-release oral morphine prescription dispensed.
Prescriptions for non-OAT opioids prior to index	NMS	Defined as any prescribed opioid dispensed excluding OAT (defined above).
Prescriptions for stimulants prior to index	NMS	Defined as any prescriptions for stimulants dispensed.
Prescriptions for benzodiazepines prior to index	NMS	Defined as any prescriptions for benzodiazepines dispensed.

Number of outpatient physician visits (excluding OUD-related visit)	OHIP	Number of OHIP billings overall in the year prior to index, excluding the following billings related to OUD.<br>OHIP feecodes excluded: A957, G040, G041, G042, G043, K682, K683, K684, K680.
Number of ED visits	NACRS	Number of unique ED visits in the year prior to index date. All ED visits are considered, regardless of diagnoses or procedures identified.
Number of inpatient hospitalizations	DAD	Number of unique ED visits in the year prior to index date. All ED visits are considered, regardless of diagnoses or procedures identified.
**RESIDENTIAL TREATMENT CHARACTERISTICS**		
Presenting problem substance (excluding opioid)	DATIS	Captures other presenting substances of concern listed at intake. Pre-defined categories reported include: Stimulants, Alcohol, Cannabis, Tobacco, Benzodiazepines, Barbiturates.
Mandatory/required Treatment	DATIS	Binary variable (yes/no) to indicate whether initiation of treatment was mandated at initiation (e.g., whether the client was mandated or ordered by a court or legal authority or an explicit condition of personal relationships, school or employment to seek treatment at an agency). Grouped using the following predefined categories: None, choice between treatment or jail, condition of probation/parole, child protective services, condition of employment, Condition of school, condition of family, other.
Reason for discharge	DATIS	Indicates primary reason residential treatment cessation and discharge. Grouped using the following predefined categories: Completed service plan, client withdrew and notified staff, mutually agreed upon, discharged by staff/involuntary discharge, transferred elsewhere (i.e., other than hospitalized), incarcerated, deceased, drop out/no show, hospitalized, other, unknown.
Length of stay	DATIS	Calculated using the date of discharge – date of admission to residential treatment (adjusted to 1 day if equal)
Services provided before/after residential treatment	DATIS	Captures other bed-based service type received prior to residential treatment. Pre-define categories reported include: withdrawal management services, residential supportive treatment, and medical/ psychiatric treatment.
**OUTCOME**		
Opioid toxicity-related ED visits and hospitalizations	DAD, NACRS	Defined as ED visits or inpatient hospitalization related to an opioid toxicity.<br><br>ICD-10 codes:

- T40.0: Poisoning by, adverse effect of and underdosing of opium
- T40.1: Poisoning by and adverse effect of heroin
- T40.2: Poisoning by, adverse effect of and underdosing of other opioids
- T40.3: Poisoning by, adverse effect of and underdosing of methadone
- T40.4: Poisoning by, adverse effect of and underdosing of other synthetic narcotic
- T40.6: Poisoning by, adverse effect of and underdosing of other and unspecified narcotics

Definitions: DAD – Discharge Abstract Database; DATIS – Drug and Alcohol Treatment Information System; DDARD – Drug and Drug/Alcohol Related Deaths; NACRS – National Ambulatory Care Reporting System; NMS – Narcotics Monitoring System; ODB – Ontario Drug Benefit database; OHIP – Ontario Health Insurance Plan database; OMHRS – Ontario Mental Health Reporting System; RPDB – Registered Persons DataBase.

References:

1. Framework MHaAP, Research Team. Mental Health and Addictions System Performance in Ontario: 2021 Scorecard Toronto, ON: ICES; 2021 [cited 2025 Jun 30]. Available from: https://www.ices.on.ca/publications/research-reports/mental-health-and-addictions-system-performance-in-ontario-2021-scorecard/.
