# Supplementary material for: Admissions to publicly-funded residential treatment for opioid use disorder and opioid toxicity outcomes after discharge in Ontario, Canada: A population-based repeated cross-sectional study
Source: PLOS Glob Public Health. 2026 Nov 11;6(11):e0007566. doi: 10.1371/journal.pgph.0007566 (PMC13754215; doi:10.1371/journal.pgph.0007566)
Supplement: Supplementary file 3 [file pgph.0007566.s003.pdf]

S3 Table. Annual counts and population adjusted rates of all residential treatment admissions for OUD (N=9,948) between July 1, 2013 and June 30, 2022.

**Year**	**Ontario population**	**N**	**Rate** (per 1,000,000 population)
2013	11,347,921	504[a]	88.8[a]
2014	11,440,138	1,310	114.5
2015	11,547,545	1,274	110.3
2016	11,684,253	1,311	112.2
2017	11,806,918	1,237	104.8
2018	11,953,757	1,210	101.2
2019	12,129,726	1,016	83.8
2020	12,310,826	700	56.9
2021	12,469,674	907	72.7
2022	12,677,785	479[a]	75.6[a]

[a] Represents 6-month count of residential treatment admissions in 2013 (Jul-Dec) and 2022 (Jan-Jun).
[b] Estimated annualized rate based on 6-month count of residential treatment admissions in 2013 (Jul-Dec) and 2022 (Jan-Jun).
