# Supplementary material for: Admissions to publicly-funded residential treatment for opioid use disorder and opioid toxicity outcomes after discharge in Ontario, Canada: A population-based repeated cross-sectional study
Source: PLOS Glob Public Health. 2026 Nov 11;6(11):e0007566. doi: 10.1371/journal.pgph.0007566 (PMC13754215; doi:10.1371/journal.pgph.0007566)
Supplement: Supplementary file 4 [file pgph.0007566.s004.pdf]

S4 Table. Estimated monthly percent changes (MPC) of all residential treatment admissions for OUD (N=9,948) between July 1, 2013 and June 30, 2022.

**Segment**	**Lower Endpoint**	**Higher Endpoint**	**MPC** (95% CI)	**P-value**
1	July 2013	September 2013	47.5 (-23.6, 184.6)	0.24
2	September 2013	January 2020	-0.3 (-0.5, -0.1)	0.0009
3	January 2020	April 2020	-20.3 (-55.2, 41.8)	0.44
4	April 2020	June 2022	2.5 (1.2, 3.7)	0.0001
