# Supplementary material for: Admissions to publicly-funded residential treatment for opioid use disorder and opioid toxicity outcomes after discharge in Ontario, Canada: A population-based repeated cross-sectional study
Source: PLOS Glob Public Health. 2026 Nov 11;6(11):e0007566. doi: 10.1371/journal.pgph.0007566 (PMC13754215; doi:10.1371/journal.pgph.0007566)
Supplement: Supplementary file 5 [file pgph.0007566.s005.pdf]

S5 Table. Annual totals and population adjusted rates of residential treatment admissions among individuals discharged from a residential treatment episode for OUD (N=9,388) between July 1st, 2013 and June 30th, 2022, overall and stratified by whether OAT was received in the 7 days following discharge from residential treatment.

		All episodes		No OAT after treatment discharge		OAT after treatment discharge	
**Year**	**Ontario population**	**N**	**Rate** (per 1,000,000 population)	**N**	**Rate** (per 1,000,000 population)	**N**	**Rate** (per 1,000,000 population)
2013	11,347,921	498[a]	87.8[b]	434[a]	76.5[b]	64	11.3[b]
2014	11,440,138	1,266	110.7	1,049	91.7	217	19.0
2015	11,547,545	1,192	103.2	942	81.6	250	21.6
2016	11,684,253	1,239	106.0	920	78.7	319	27.3
2017	11,806,918	1,177	99.7	855	72.4	322	27.3
2018	11,953,757	1,141	95.5	772	64.6	369	30.9
2019	12,129,726	896	73.9	566	46.7	330	27.2
2020	12,310,826	673	54.7	367	29.8	306	24.9
2021	12,469,674	858	68.8	441	35.4	417	33.4
2022	12,677,785	448[a]	70.7[b]	218[a]	34.4[b]	230	36.2[b]

[a] Represents 6-month count of residential treatment admissions in 2013 (Jul-Dec) and 2022 (Jan-Jun).
[b] Estimated annualized rate based on 6-month count of residential treatment admissions in 2013 (Jul-Dec) and 2022 (Jan-Jun).
