# Supplementary material for: Admissions to publicly-funded residential treatment for opioid use disorder and opioid toxicity outcomes after discharge in Ontario, Canada: A population-based repeated cross-sectional study
Source: PLOS Glob Public Health. 2026 Nov 11;6(11):e0007566. doi: 10.1371/journal.pgph.0007566 (PMC13754215; doi:10.1371/journal.pgph.0007566)

S1 Figure. Monthly population adjusted rates of residential treatment admissions among individuals discharged from a residential treatment episode for OUD (N=9,388) between July 1st, 2013 and June 30th, 2022, overall and stratified by whether OAT was received in the 7 days following discharge from residential treatment.

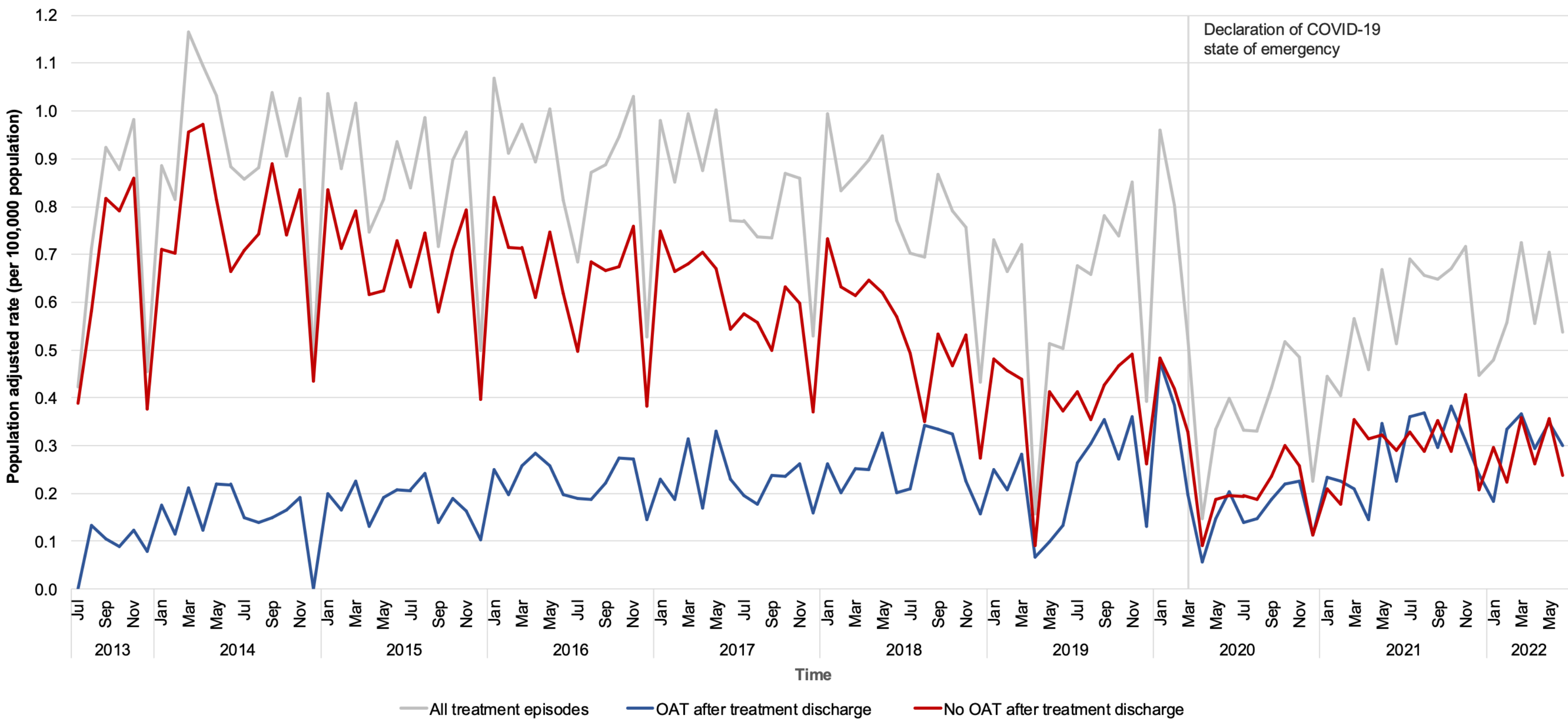

Supplement: Supplementary file 6 [file pgph.0007566.s006.pdf]
